# Cholangiolocellular Carcinoma Misdiagnosed As Hemangioma: Unique Central Fibrotic Area Devoid of Cancer Cells Detected on a Seven-Year Follow-Up

**DOI:** 10.7759/cureus.73091

**Published:** 2024-11-05

**Authors:** Wenhua Shao, Hitomi Kamo, Kazuo Yoshioka, Keisuke Izumi, Koichi Tsuneyama

**Affiliations:** 1 Department of Molecular Pathology, Tokushima University, Tokushima, JPN; 2 Department of Surgery, Taoka Hospital, Tokushima, JPN; 3 Department of Pathology and Laboratory Medicine, Tokushima University, Tokushima, JPN

**Keywords:** central fibrous area, cholangiocarcinoma, cholangiolocellular carcinoma, hemangioma, long-term follow-up

## Abstract

Cholangiolocellular carcinoma, a subtype of peripheral-type intrahepatic cholangiocarcinoma, is a relatively rare primary liver tumor. This case report describes a patient with cholangiolocellular carcinoma that was initially misdiagnosed as hemangioma, and ultimately underwent complete tumor resection after a seven-year follow-up period. A 72-year-old female patient with a history of chronic hepatitis C was followed up regularly at the hospital. Computed tomography (CT) performed seven years prior had detected a small tumor (12 mm) with radiographic characteristics suggestive of hemangioma. The tumor increased in size from 12 to 32 mm over the next seven years. On CT, the tumor showed poor central enhancement, indicating reduced blood flow in the central region. Due to the suspicion of malignancy, partial surgical resection was performed. Pathological examination confirmed a diagnosis of cholangiolocellular carcinoma. The carcinoma exhibited vascular invasion and a broad central fibrous area with hyalinization, lacking epithelial cells, and marked by venous obstruction. This case underscores the challenge of distinguishing cholangiolocellular carcinoma from hemangioma, particularly in small tumors with similar radiological features. The findings highlight the importance of employing additional diagnostic modalities such as ultrasound and magnetic resonance imaging, as well as the necessity of biopsy when suspicion arises. This case also describes the unique finding of a central fibrous area without tumor cells, which may have resulted from localized circulatory disturbances potentially caused by tumor embolism in the portal and hepatic veins. This pathological finding provides valuable insights into the nature of this rare tumor.

## Introduction

Cholangiolocellular carcinoma, particularly in its pure and small-sized form, is characterized by a relatively low fibrous component and may exhibit medullary growth patterns. This can result in poor enhancement on imaging modalities performed initially, such as dynamic CT. In addition, these tumors may present with a hemangioma-like appearance due to the migration of contrast medium from the portal vein to the interstitial tissue during the equilibrium phase [1]. Cholangiolocellular carcinomas often exhibit slower growth rates compared to typical hepatocellular carcinomas, with minimal size changes over the course of one year. The slow progression can lead to misdiagnosis as "hemangioma" and result in routine follow-up without further investigation [2-4].

We encountered the case of a patient who was followed up for seven years with a presumed diagnosis of hemangioma, in whom the tumor was later surgically removed and confirmed to be cholangiolocellular carcinoma (a peripheral type of intrahepatic cholangiocarcinoma).

## Case presentation

**Figure 1:**
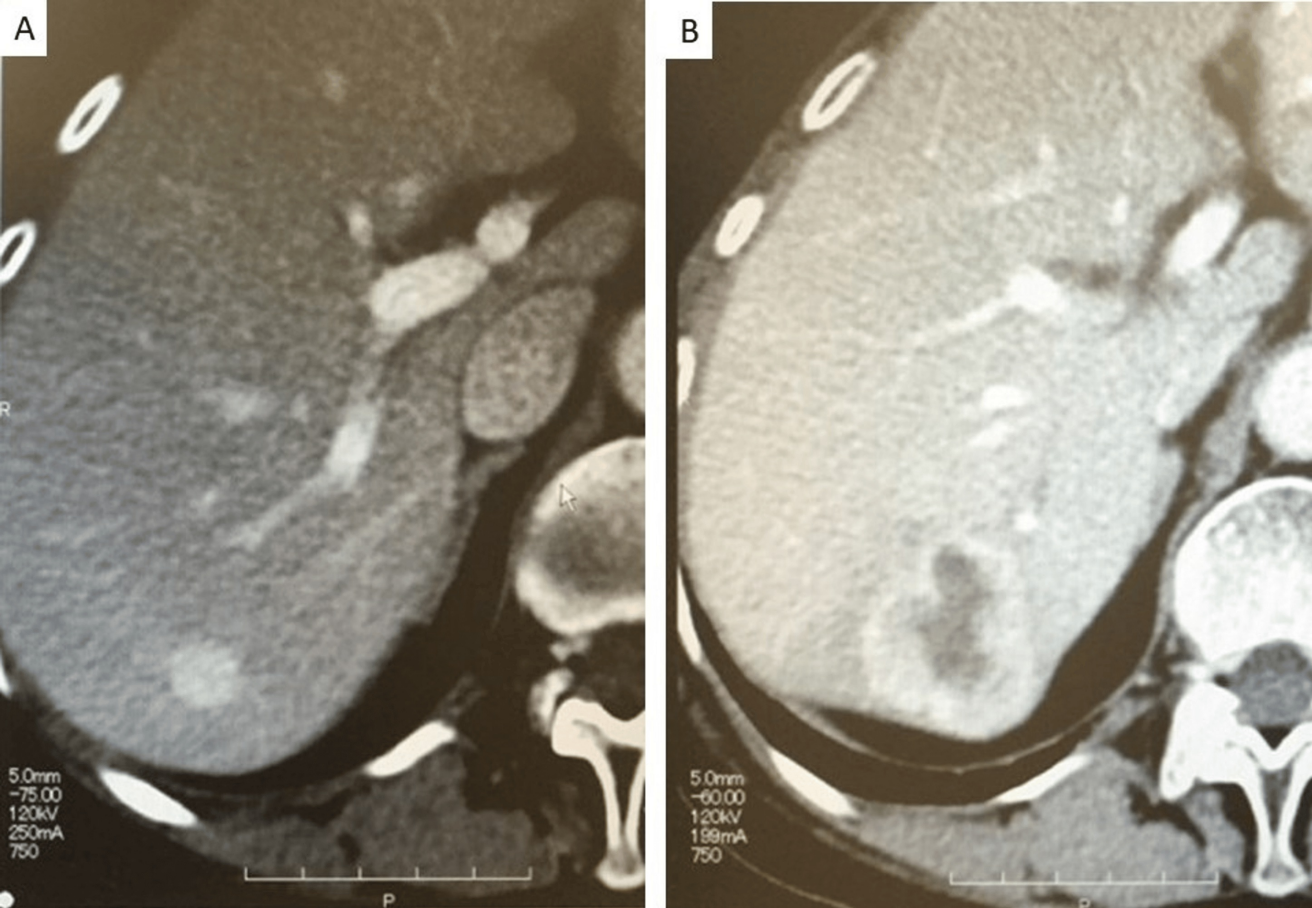
Contrast-enhanced CT images; (A) seven years prior, and (B) current presentation. A: A 12-mm tumor with uniform contrast enhancement is seen in the liver. B: The tumor diameter has increased to 35 mm, with irregular margins and minimal internal contrast, suggesting vascular attenuation.

A liver tumor was detected seven years previously during a routine checkup in a 72-year-old female patient with chronic hepatitis C. Computed tomography (CT) performed at that time identified a well-defined tumor measuring 12 mm in diameter. Contrast-enhanced CT showed slight tumoral enhancement in the arterial phase that intensified in the portal venous phase. The tumor was isodense to normal parenchyma in the equilibrium phase, suggesting a diagnosis of typical hemangioma. As the tumor was considered benign, no further procedures were performed. Regular follow-up continued for the first few years, but as the size and nature of the tumor had remained unchanged over this period, the patient was not followed up with imaging in the most recent three years. CT obtained seven years after the initial detection showed an increase in diameter to 35 mm with poor central enhancement, suggesting decreased blood flow in the central region of the tumor (Figure 1). Given the suspicion of malignancy, partial surgical resection was performed.

**Figure 2:**
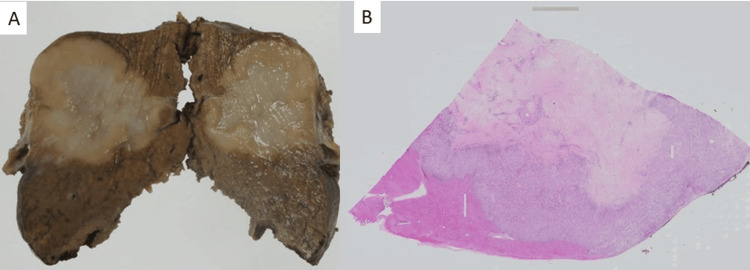
Gross appearance (A) and loupe image (B) of the surgical specimen. A: The tumor measures 38 × 32 mm and has well-defined borders. The margins are yellowish, while the center appears whitish. B: The central area of the tumor is fibrous, with cellular components visible at the periphery.

A well-defined tumor measuring 38 × 32 mm was found in contact with the liver capsule, located 12 mm from the margin. The tumor exhibited a distinctly different color compared to normal parenchyma, with a whitish center and yellowish edges. The tumor was completely excised (Figure 2A). Histologically, the differently colored areas of the tumor showed marked differences: the central white area consisted of dense collagenous tissue with hyalinization and few cellular components, whereas the yellowish area at the periphery was rich in cellular components (Figure 2B).

**Figure 3:**
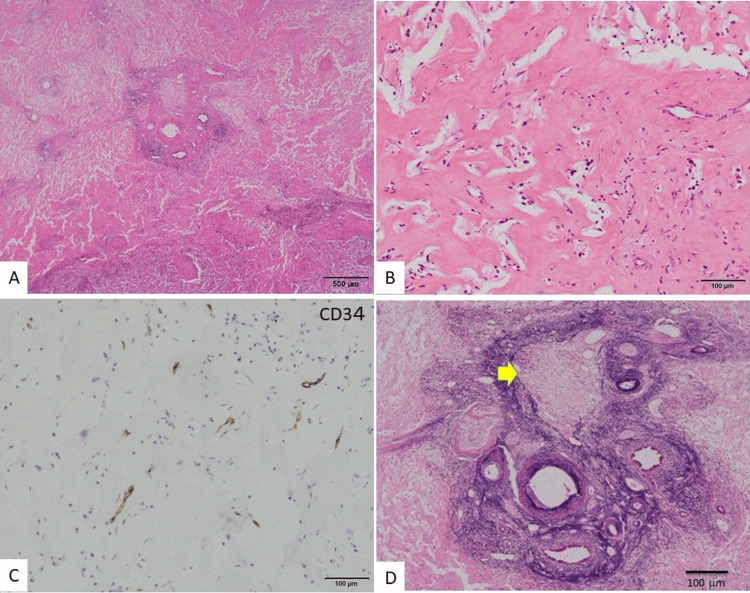
Histological images of the fibrous area in the center of the tumor. A, B: Hematoxylin and eosin (HE) staining. Epithelial cells, including hepatocytes, were not observed, though a few portal areas remained. The fibrous tissue showed hyalinization without the anastomosing structures typical of hemangiomas. C: Immunostaining of CD34, a vascular endothelial cell marker, was positive only in scant capillary vessels. D: Victoria blue with HE staining. Complete occlusion or slight recanalization of the portal vein was observed (arrow). Scale bar: A, 500 μm; B, C, D, 100 μm.

The central area exhibited dense fibrous tissue with hyalinization and scant vascularity. Although no epithelial cells (including hepatocytes) were observed, remnants of a few portal areas were seen (Figure 3A). The dense fibrous area contained no anastomosing structure typically seen in hemangiomas (Figure 3B). CD34, a vascular endothelial cell marker, was positive in only a few capillary vessels (Figure 3C). Complete occlusion with slight recanalization of the portal vein and hepatic venules was observed sporadically (Figure 3D).

**Figure 4:**
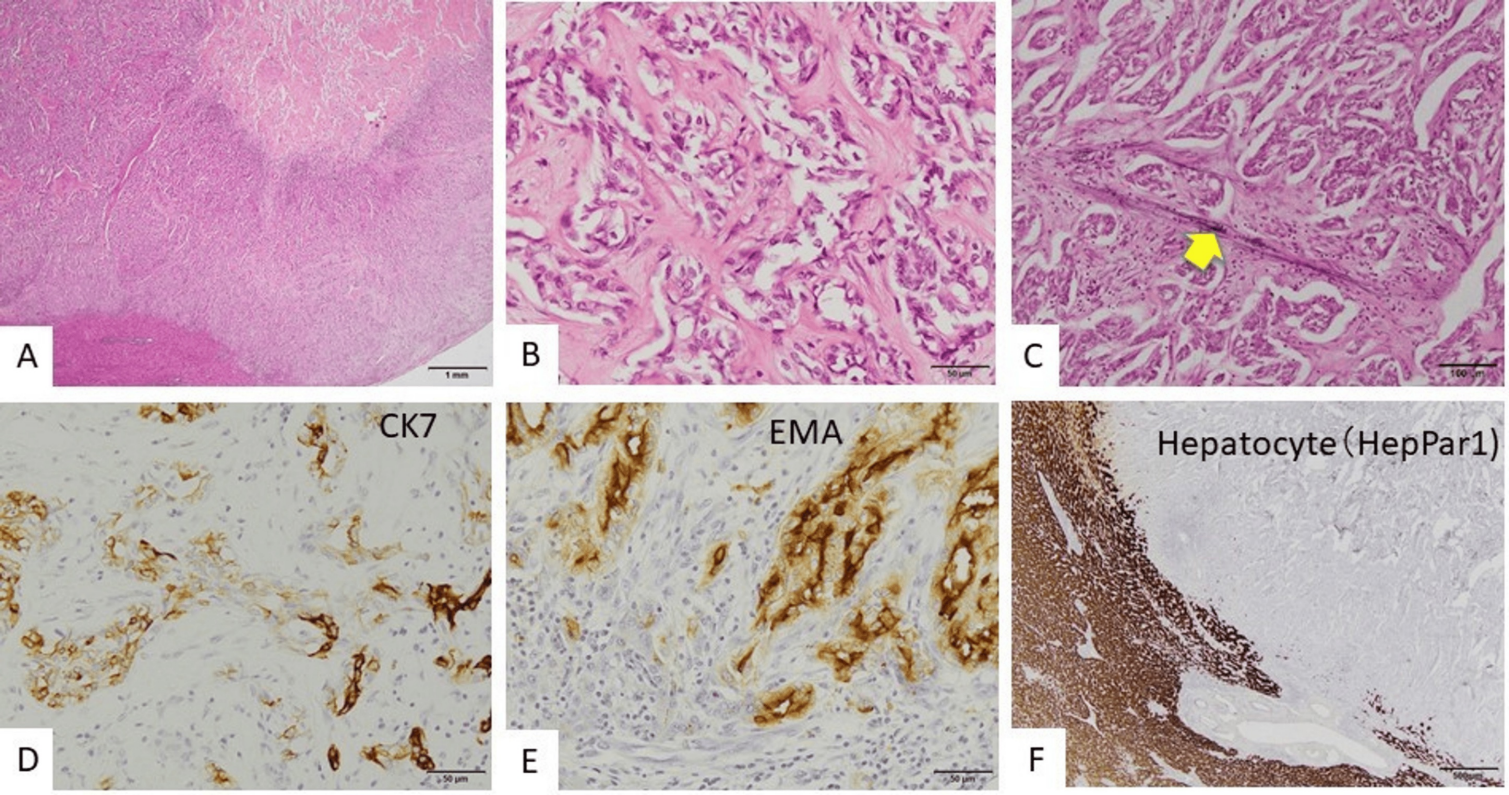
Images of the periphery of the tumor A, B: Hematoxylin and eosin (HE) staining. Tumor cells with enlarged, irregularly shaped nuclei demonstrate ductal branching or trabecular formation amidst fibrous proliferation. C: Victoria blue with HE staining. Vascular invasion is observed (arrow). D–F: Immunostaining (D: CK7, E: EMA, F: Hepatocyte). Tumor cells show strong or weak CK7 positivity in the cytoplasm. EMA (Epithelial Membrane Antigen) staining is more intense on the luminal surface of the tumoral cords. Hepatocytes are negative for tumor markers. Scale bar: A, 1 mm; B, D, E, 50 μm; C, 100 μm; F, 500 μm.

In the peripheral area of the tumor, cells appeared as ductal formations with complex branching or trabecular structures, along with fibroplasia and enlarged, irregularly shaped nuclei (Figures 4A, 4B). Occasional vascular invasion by tumor cells was observed (Figure 4C). The cytoplasm of some tumor cells was positive for CK7 (Figure 4D). Interestingly, EMA (Epithelial Membrane Antigen) staining was most intense on the luminal surface of the tumoral cords (Figure 4E). Hepatocyte (HepPar1)-positive cells were not observed in the tumor (Figure 4F). The background liver showed moderate lymphocytic infiltration in portal areas with mild interface hepatitis. The fibrous septa were mildly elongated and mild necro-inflammatory changes in the liver parenchyma were suggestive of chronic hepatitis, which was staged as A1, F1. Based on these findings, we concluded that cholangiolocellular carcinoma (a peripheral type of intrahepatic cholangiocarcinoma) had developed in the context of chronic hepatitis.

The patient was followed up as an outpatient after surgery. However, five years and two months after the operation, a recurrence was detected at the resection margin, and TACE (transarterial chemoembolization with Lipiodol and epirubicin) was performed by the Department of Interventional Radiology, Taoka Hospital, Tokushima. Two years later, another recurrence occurred near the edge of the tumor, and TACE (intra-arterial chemotherapy with Lipiodol and epirubicin) was administered again. There has been no recurrence for the past two years.

## Discussion

In patients with chronic liver disease, who are at high risk for hepatocellular carcinoma, cholangiolocellular carcinoma can be suspected based on the findings of dynamic CT performed across four phases (unenhanced, arterial, portal, and equilibrium). However, cholangiolocellular carcinoma often occurs without underlying chronic liver disease, leading to an imaging diagnosis of "suspected hemangioma" that may remain unchanged on follow-up at three to six months [1]. The present case highlights the critical importance of utilizing additional diagnostic modalities such as ultrasound and magnetic resonance imaging (MRI), and ultimately confirming the diagnosis through biopsy when there is even slight suspicion of malignancy.

A unique aspect of this case is that it enables observation of imaging changes in cholangiolocellular carcinoma over a seven-year period, revealing the formation of an extensive fibrous stroma with hyalinization in the center of the resected tumor, devoid of any tumor cells. Although cholangiolocellular carcinoma is typically characterized by the presence of abundant fibrous stroma, cases with extensive fibrotic regions and a sparse cellular component and no cancer cells within the tumor, as observed here, are rarely reported [5]. Our patient was monitored without intervention under the assumption of hemangioma, and no treatment modifications were made. The findings of serial CT suggest that a specific pathological event may have occurred during tumor progression, leading to formation of the distinctive fibrotic areas.

Remnants of portal areas were evident within the fibrotic regions, along with signs of portal vein occlusion. In addition, tumor emboli were detected within veins located at the tumor margins, in areas of viable tumor growth. We propose that in this case, the central fibrotic area devoid of tumor cells may have resulted from a localized circulatory disturbance that was potentially initiated by tumor emboli in the portal and hepatic veins. This unique pathological finding was clearly visualized on imaging and is a meaningful contribution to our understanding of the underlying nature of cholangiolocellular carcinoma.

## Conclusions

This case underscores the challenge of distinguishing cholangiolocellular carcinoma from hemangioma, particularly in small tumors with similar radiological features. The findings highlight the importance of employing additional diagnostic modalities, such as ultrasound and MRI, and the necessity of biopsy when suspicion arises. The case also reveals a unique central fibrous area without tumor cells, which may result from localized circulatory disturbances potentially caused by tumor embolism in the portal and hepatic veins. This pathological finding provides valuable insights into the nature of this rare tumor.

## References

[1] Motosugi U, Ichikawa T, Nakajima H (2009). Cholangiolocellular carcinoma of the liver: imaging findings. J Comput Assist Tomogr.

[2] Akiyama K, Abe T, Oshita A (2021). Gradually progressive cholangiolocellular carcinoma: a case report. Surg Case Rep.

[3] Yamaguchi K, Nagao Y, Yamane S (2018). A case of cholangiolocellular carcinoma found to be hepatic hemangioma at 16-month follow up (Article in Japanese). Nihon Shokakibyo Gakkai Zasshi.

[4] Koga Y, Nagahama H, Tateyama M (2012). A case of cholangiolocellular carcinoma combined with intrahepatic cholangiocarcinoma diagnosed after 4 years follow-up for hepatic hemangioma (Article in Japanese). Nihon Shokakibyo Gakkai Zasshi.

[5] Kim T, Hori M, Onishi H (2009). Liver masses with central or eccentric scar. Semin Ultrasound CT MR.

